# Optimization of a Luciferase-Expressing Non-Invasive Intrapleural Model of Malignant Mesothelioma in Immunocompetent Mice

**DOI:** 10.3390/cancers12082136

**Published:** 2020-08-01

**Authors:** Elisabeth Digifico, Marco Erreni, Federico Simone Colombo, Camilla Recordati, Roberta Migliore, Roberta Frapolli, Maurizio D’Incalci, Cristina Belgiovine, Paola Allavena

**Affiliations:** 1 Laboratory of Cellular Immunology, Humanitas Clinical and Research Center - IRCCS -, Via Manzoni 56, 20089 Rozzano (MI), Italy; elisabeth.digifico@humanitasresearch.it (E.D.); roberta.migliore91@gmail.com (R.M.); 2 Unit of Advanced Optical Microscopy, Humanitas Clinical and Research Center - IRCCS -, Via Manzoni 56, 20089 Rozzano (MI), Italy; marco.erreni@humanitasresearch.it; 3 Flow Cytometry and Cell Sorting Unit, Humanitas Clinical and Research Center - IRCCS -, Via Manzoni 56, 20089 Rozzano (MI), Italy; Federico_Simone.Colombo@humanitasresearch.it; 4 Department of Veterinary Medicine, University of Milan, 26900 Lodi, Italy; camilla.recordati@unimi.it; 5 Department of Oncology, Mario Negri Institute for Pharmacological Research - IRCCS -, 20156 Milan, Italy; roberta.frapolli@marionegri.it (R.F.); maurizio.dincalci@marionegri.it (M.D.)

**Keywords:** malignant pleural mesothelioma, MPM mouse model, in vivo imaging, intrathoracic injection, inflammation, tumor microenvironment

## Abstract

Malignant Pleural Mesothelioma (MPM) is an aggressive tumor of the pleural lining that is usually identified at advanced stages and resistant to current therapies. Appropriate pre-clinical mouse tumor models are of pivotal importance to study its biology. Usually, tumor cells have been injected intraperitoneally or subcutaneously. Using three available murine mesothelioma cell lines with different histotypes (sarcomatoid, biphasic, epithelioid), we have set up a simplified model of in vivo growth orthotopically by inoculating tumor cells directly in the thorax with a minimally invasive procedure. Mesothelioma tumors grew along the pleura and spread on the superficial areas of the lungs, but no masses were found outside the thoracic cavity. As observed in human MPM, tumors were highly infiltrated by macrophages and T cells. The luciferase-expressing cells can be visualized in vivo by bioluminescent optical imaging to precisely quantify tumor growth over time. Notably, the bioluminescence signal detected in vivo correctly matched the tumor burden quantified with classical histology. In contrast, the subcutaneous or intraperitoneal growth of these mesothelioma cells was considered either non-representative of the human disease or unreliable to precisely quantify tumor load. Our non-invasive in vivo model of mesothelioma is simple and reproducible, and it reliably recapitulates the human disease.

## 1. Introduction

Malignant Mesothelioma is an aggressive and rare cancer arising from the mesothelial cells that line the pleura, the peritoneum, or the pericardial cavity. Eighty percent of the cases are of pleural origin and have been defined as Malignant Pleural Mesothelioma (MPM) [1]. Mesothelioma incidence is strongly related to occupational or environmental exposure to airborne asbestos, with 1400 new cases/year in Italy. It is characterized by a non-resolving, long-lasting inflammation driven by the presence of non-degradable asbestos fibers [2,3]. Although asbestos use and production was banned in Italy in 1992, and its use is restricted in several Western countries, MPM incidence is still growing, and the peak is expected around 2020–2025.

MPM has a very long latency period, up to 20–30 years, and it is usually identified at advance stages, because biomarkers and radiological diagnostic tools are not effective for its early detection [4]. Chronic inflammation has been recognized as the first pathogenic step in the long chain of events that drives the development of mesothelioma. Asbestos fibers in the lungs generate a long-lasting reactive inflammation in macrophages and in mesothelial cells, which is non-resolving, due to the undegradable nature of these fibers. Over several years, this chronic inflammation causes DNA damage, the accumulation of DNA mutations, and finally neoplastic transformation.

Human MPM tumors have been histologically classified into three main histotypes, based on the morphology of the tumor cells: epithelioid, sarcomatoid, and biphasic. The epithelioid histotype represents 60% of all MPM, and it is usually associated with the longest survival (12–27 months), while the sarcomatoid is the least common histotype (20%), but it is associated with the worst patient survival (7–18 months); biphasic MPM is characterized by a combination of cells with both epithelioid and sarcomatoid morphology and has an intermediate behavior (8–21 months) [5].

MPM is dramatically resistant to conventional therapies such as radiation and chemotherapy. Checkpoint immunotherapy is under investigation in preclinic with, so far, non-optimal results. Thus, more effective treatments are urgently needed [6,7,8,9].

Historically, to study mesothelioma tumorigenesis, mice were intraperitoneally or intrapleurally injected with asbestos fibers. Even if close to the human pathogenetic conditions, this procedure required a long latency period to progress in full carcinogenesis (7–25 months), and only some mice developed tumors (about 35%) [10]. Moreover, the injection of asbestos fibers entails a high risk for the operator, who must handle them following strict safety procedures. From these difficult models, some murine mesothelioma cells were isolated and cultured in vitro as continuously growing mesothelioma cell lines [10,11,12]. These established cell lines are ideal tools for studying in vivo MPM tumor development. In most reports, MPM have been transplanted intraperitoneally, subcutaneously, or directly in the thorax of mice, the latter procedure involving a surgical resection, which may be challenging and frequently associated with some mortality [13,14,15].

Subcutaneous growth of MPM is not an orthotopic model and therefore not suitable to study its biology. On the other hand, in the intraperitoneal model (most commonly used), tumor cells do not grow only on the mesothelial lining but also around the intraperitoneal organs in an unpredictable manner.

We present here the optimization of an orthotopic model of murine MPM with direct intrathorax injection of tumor cells with a minimally invasive procedure. The use of luciferase-expressing tumor cells has been exploited to successfully and reproducibly quantify tumor growth in vivo along time.

## 2. Results

### 2.1. In Vivo Growth of Murine Mesothelioma Tumors and Characterization of the Immune Infiltrate

**Figure 1:**
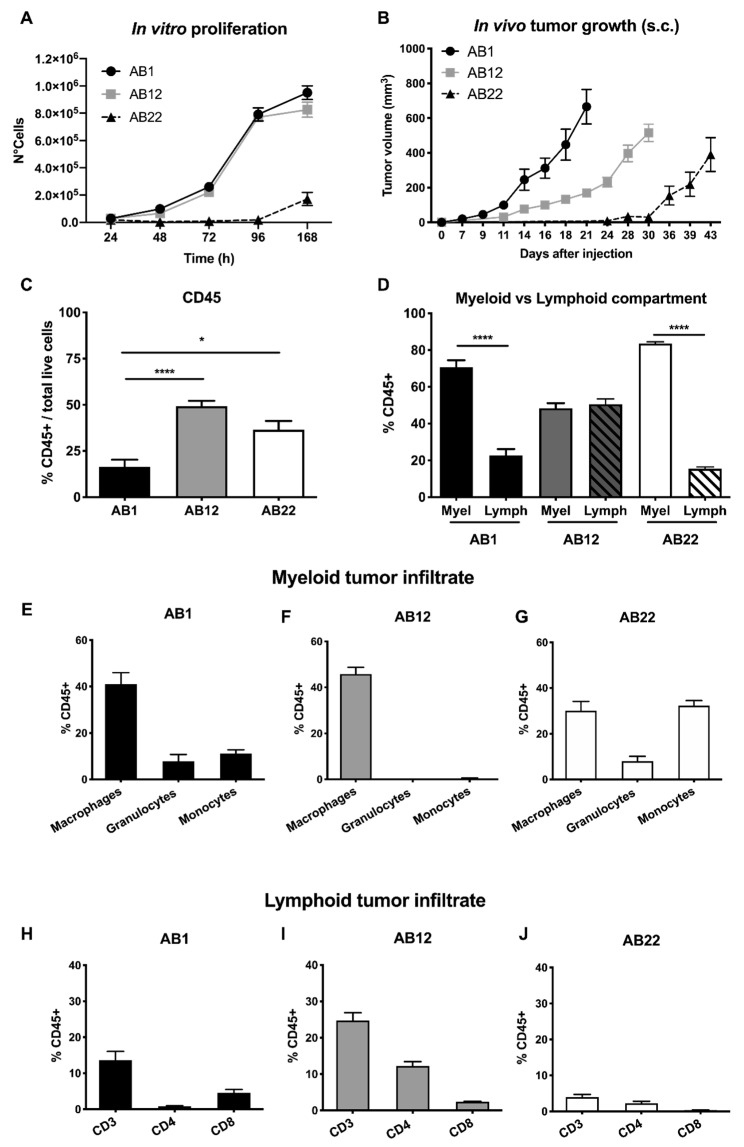
Subcutaneous mesothelioma tumor growth. (**A**) In vitro growth of murine mesothelioma cell lines. AB1 and AB12 cells have a higher in vitro proliferation rate compared with AB22. (**B**) The tumor growth curve of AB1, AB12, and AB22 injected subcutaneously (s.c.) in BALB/c mice. Data are expressed as mean of tumor volume (mm^3^) ± SEM. (**C**–**J**) Flow cytometry of the leukocyte infiltrate in the subcutaneous tumor masses. (**C**) Percentage of CD45^+^ cells over total cells alive from tumor cell suspension. (**D**) AB1 (sarcomatoid) and AB22 (epithelioid) mesothelioma are richer in the myeloid compartment compared to the lymphoid one. AB12 tumors (mixed phenotype) have similar representation of the two compartments. (**E–J**) Percentage (over CD45^+^ cells) of different myeloid and lymphoid subset in tumor derived from the three cell lines. Data are expressed as mean ± SEM. AB1: *n* = 8 mice; AB12: *n* = 5; AB22: *n* = 3. Statistical analysis: Unpaired student’s *t*-test with Welch’s correction (* *p* < 0.05, **** *p* < 0.0001).

The murine mesothelioma cell lines AB1, AB12, and AB22, have been previously generated in BALB/c mice upon intraperitoneal injection of crocidolite asbestos fibers [10]. Of note, these three cell lines recapitulate the three human histotypes of mesothelioma: AB1 cells have a sarcomatoid morphology, AB22 is epithelioid, and AB12 has a biphasic phenotype [11]. Their in vitro growth shows a linear rate of proliferation over time and a slower rate for the epithelioid AB22 cell line (Figure 1A). Next, we investigated the in vivo tumor growth of these murine mesothelioma cell lines. In a first series of experiments, cells were injected subcutaneously (1 × 10^5^ cells) in BALB/c mice, and tumor growth was measured by caliper over time. AB1 cell-derived tumors became measurable 7–9 days post injection, showing a faster tumor growth compared to AB22 cell-derived tumors, which appeared only 24–28 days after administration; AB12 tumors had an intermediated behavior (Figure 1B). When mice reached the ethical endpoint (loss of >10% of body weight), they were sacrificed, around day 21, 30, and 43 for AB1, AB12, and AB22, respectively. Explanted tumor masses were enzymatically dissociated and analyzed to characterize the immune infiltrate by flow cytometry. The gating strategy is presented in Supplementary Figure S1. Interestingly, AB1 (sarcomatoid) and AB22 (epithelioid)-derived tumors were richer in the myeloid compartment (up to 70–80% of CD11b^+^ cells/total CD45^+^ cells), (Figure 1D,E,G,H,J). In contrast, tumors from AB12 cells (mixed histotype) were equally infiltrated by myeloid and lymphoid cells, even if the first represented only by macrophages (Figure 1F,I).

**Figure 2:**
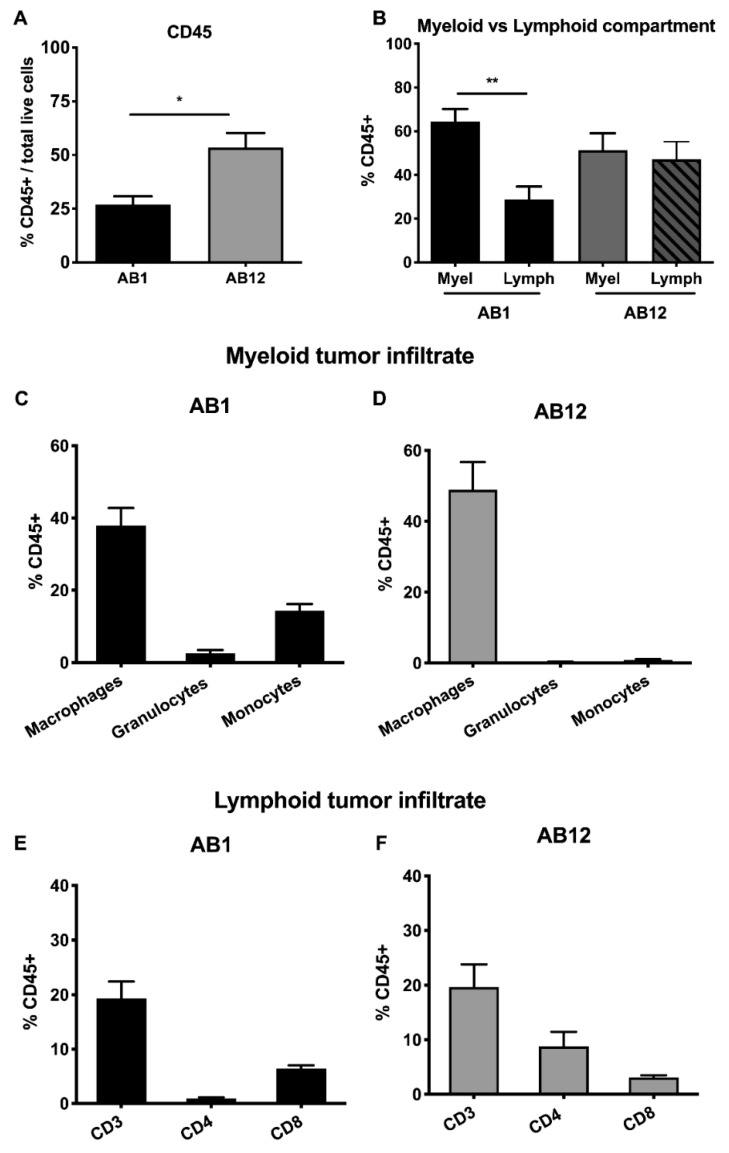
Intraperitoneal mesothelioma tumor growth. Flow cytometry of the leukocyte infiltrate in the intraperitoneal tumor masses. (**A**) Percentage of CD45^+^ cells over total cells alive from tumor cell suspension. (**B**) AB1 (sarcomatoid) mesothelioma tumors are richer in the myeloid compartment compared to the lymphoid one. AB12 tumors (mixed phenotype) have similar representation of the two compartments. (**C–F**) Percentage (over CD45^+^ cells) of different myeloid and lymphoid subset in tumor derived from AB1 and AB12 cell lines. Data are expressed as mean ± SEM. AB1: *n* = 7 mice; AB12: *n* = 5. Statistical analysis: Unpaired student’s *t*-test with Welch’s correction (* *p* < 0.05, ** *p* < 0.01).

In a parallel experiment, we injected mesothelioma cells intraperitoneally (1 × 10^5^ cells) in BALB/c mice. AB1 and AB12 cells grew in the peritoneum; however, it was not possible to monitor tumor growth over time; at sacrifice, numerous small tumor masses were visible all over the surface of the peritoneum and abdominal organs. These masses were collected as much as possible, but a reliable quantification was not possible. For AB22 cells, only few or no masses were recovered from the peritoneal cavity. The flow cytometry analysis of the dissociated tumors derived from AB1 and AB12 cells totally confirmed the results achieved in the subcutaneous model, with a predominance of myeloid cells and paucity of T cells (Figure 2A–D).

**Figure 3:**
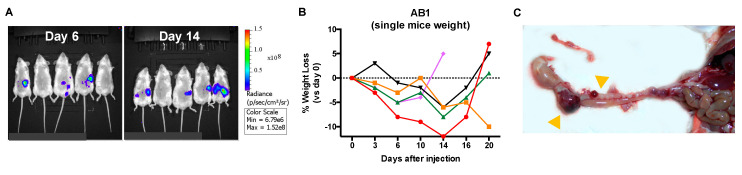
Intraperitoneal injection of the murine AB1 mesothelioma cell line: in vivo imaging. (**A**) Representative images of luciferase signal acquisition at day 6 and 14 post injection, performed by IVIS system (IVIS Lumina III, Perkin Elmer). (**B**) Percentage of weight loss over time for each mouse. (**C**) Representative picture of the chain of small tumor masses grown in the peritoneal cavity.

In a second experiment, we exploited the luciferase signal expressed by the mesothelioma cells to monitor tumor growth in vivo by quantifying bioluminescence using the IVIS system (IVIS Lumina III, Perkin Elmer). The AB1 sarcomatoid cell line was injected intraperitoneally (i.p.), and the luciferase signal was acquired along the experiment. A signal was visible as early as day 6 post injection in 3/5 mice and in 4/5 mice at day 14, but it did not appear as a linearly increasing signal over time (Figure 3A). Unexpectedly, we observed that between day 16 and 20, some mice rapidly lost considerable weight and appeared very sick (Figure 3B). At day 20, all mice were sacrificed and underwent autopsy: we noticed several little intraperitoneal masses growing as chain-like ropes along the entire abdominal cavity, from the stomach to the rectum (Figure 3C). One mouse had an intestinal block, and two mice showed an extensive hemorrhagic area.

Overall, these in vivo experiments were not satisfactory for several reasons: (1) the subcutaneous tumor model, even if easy to perform and growing in a linear mode, is not an orthotopic model and is not suitable to study the biology of mesothelioma; (2) in the intraperitoneal model, the tumor disseminates in several small masses, which are difficult to quantify. Furthermore, injected tumor cells are not growing only along the mesothelium lining, but also around the intraperitoneal organs in an unpredictable manner.

### 2.2. Setup of a New Intrapleural Murine Mesothelioma Model

**Figure 4:**
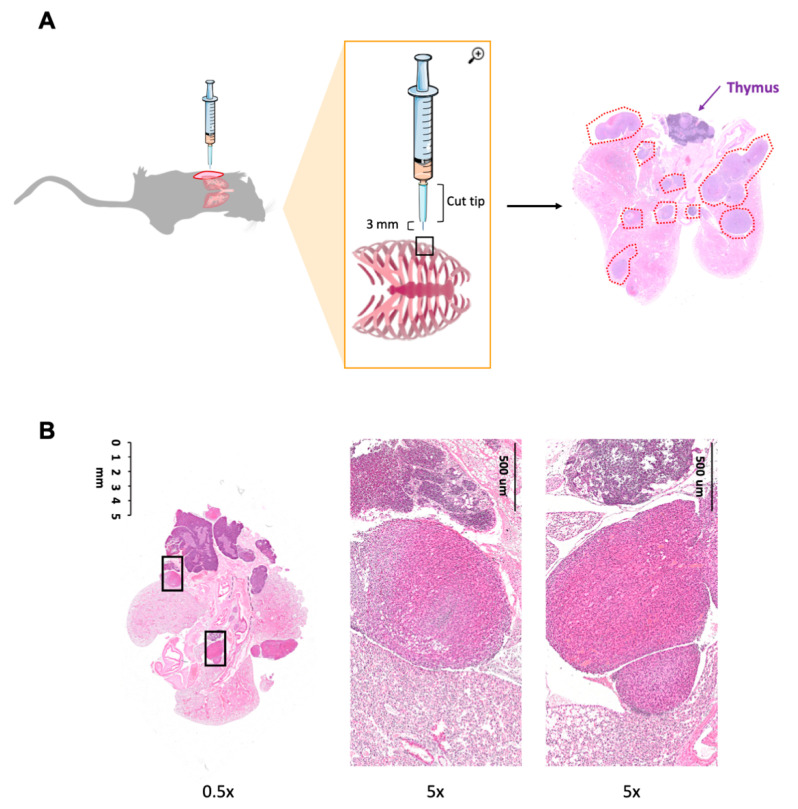
Scheme of tumor implantation of Malignant Pleural Mesothelioma (MPM) cells by intrathorax injection. (**A**) Mice were anesthetized, the skin was cut for 1 cm length and MPM cells (5 × 10^4^) were injected between the third and fourth costal space. To avoid lung perforation, the syringe (500 uL syringe) has been overmounted with a cut P200 tip. On the right and in (**B**) representative images of hematoxylin–eosin (H&E) staining of the lung; some tumor masses are visible (red dotted lines).

The fact that MPM tumors growing i.p. were not precisely measurable and that adverse events were noted in some mice (depending on which abdominal organs were affected by tumor growth) prompted us to set up a model of mesothelioma injected directly in the thorax. We optimized a minimally invasive localized injection of mesothelioma cells, as much as possible close to the pleura, as shown in Figure 4A. The skin of anesthetized mice was superficially incised between the third and the fourth costal space; mesothelioma cells (5 × 10^4^) were injected in the thoracic cavity using a needle overmounted with a cut tip (in order to have an uncovered, fixed maximum length of the needle of 3 mm, to avoid lung perforation). This procedure was very reproducible: out of 50 mice injected, the engraftment rate was 100%. Tumor growth developed over time in 94% of mice; only few (3/50) mice had detectable but very slow growing tumors (histology and in vivo imaging).

Of note, mice developed tumors without significant weight loss or evident suffering during the experiment. At autopsy, tumor nodules/masses were observed inside the thoracic cavity growing on the pleura and partially infiltrating the lobes, but no masses were ever found outside the thoracic cavity (Figure 4B).

### 2.3. In Vivo Quantification of Tumor Growth of Murine Mesothelioma in the Thorax

**Figure 5:**
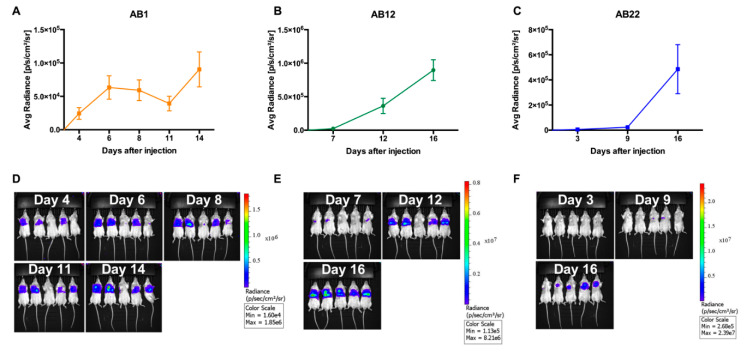
In vivo growth of 5 × 10^4^ AB1, AB12, and AB22 mesothelioma cells injected intrathoracically in BALB/c mice. (**A**) AB1 cells are the fastest and start growing at day 4-6 post injection. (**B**) AB12 cells start growing at day 7 post injection. (**C**) AB22 cells are the slowest and start growing after 9 days post injection. (**D**–**F**) IVIS acquisition of luciferase (LUC) signal at different time points. Data are expressed as mean ± SEM.

To quantify tumor burden of the in vivo grown tumors after intrathoracic injection, we measured over time the luciferase signal with IVIS. Data were analyzed with the Living Image software (v4.3.1, Perkin Elmer) by designing a ROI (region of interest) on the thoracic area of each mouse. Figure 5A–F shows representative tumor growth experiments. We confirmed that AB1 tumors (sarcomatoid) are the most aggressive, being detectable at day 4 post injection; AB22 tumors (epithelioid) grow slowly, with measurable tumors 9 days after tumor cell injection; AB12, the biphasic cell line, showed an intermediate behavior (Figure 5A–F; Supplementary Figure S2). Importantly, a linear increasing signal was detected over time. However, it should be noted that the growth curve of AB1-formed tumors is bending around day 11 post injection, and then it is growing again at later times (Figure 5 and Supplementary Figure S2). This behavior is likely the result of a process of “tumor editing” by the immune system against the cancer cells. 

**Figure 6:**
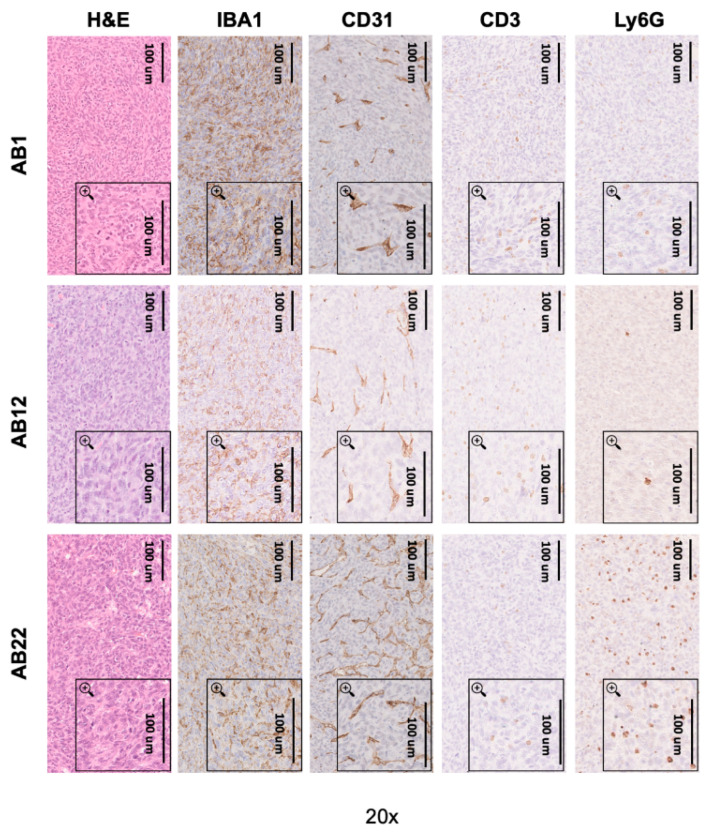
Immunohistochemistry on mouse lungs. Representative images of hematoxylin/eosin, IBA1 (macrophages), CD31 (vessels), CD3 (lymphocytes), and Ly6G (neutrophils) staining in AB1, AB12, and AB22 tumors.

At sacrifice, the explanted lungs of mice bearing tumors formed by AB1, AB12, and AB22 cells were paraffin-embedded and analyzed by immunohistochemistry. AB1, AB12, and AB22 tumors were strongly infiltrated by IBA1+ macrophages and CD31+ vessels (especially AB22), while CD3+ lymphocytes were very rare in the three tumor types (Figure 6). Surprisingly, AB22 tumors showed a higher level of neutrophil infiltration compared with AB1 and AB12 tumors.

### 2.4. Comparison of In Vivo Imaging and Classical Histology to Monitor Tumor Load in the Intrathorax Model of Murine MPM

**Figure 7:**
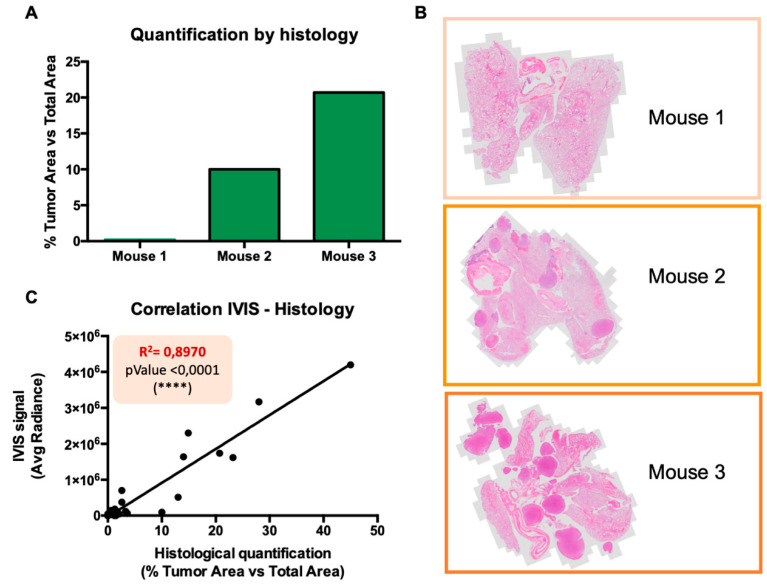
Validation of IVIS tumor measurement system. (**A**) Quantification of the tumor load (tumoral area versus total area of the lungs) in three histological sections of mouse lungs (shown in **B**). (**B**) Representative pictures of hematoxylin–eosin (H&E) staining of lungs from mice injected intrathoracically. (**C**) Correlation between the tumor quantification measured by IVIS (average radiance (p/s/cm^2^/sr)) and by histology (percentage of tumor area versus the total area of each lung) (*n* = 26 mice).

To additionally check that the intensity of the bioluminescent signal was indeed correlating with the tumor load, we stained slices of whole lungs with hematoxylin–eosin (H&E) and quantified the tumor areas relative to the total area of each lung with ImageJ v2.0 software. Figure 7A,B shows histological samples from three representative mouse lungs displaying little, medium, and large tumor load, respectively. Considering a total number of 25 mice, we observed an almost perfect correlation in tumor quantification between the bioluminescent signal detected in vivo and conventional histology performed ex vivo (Figure 7C). Therefore, we conclude that the monitoring of the bioluminescent signal is a reliable and suitable method for the in vivo quantification of mesothelioma growth in the intrathorax model.

## 3. Discussion

In this study, we have performed a full characterization of an orthotopic model of murine mesothelioma obtained with a minimally invasive intrapleural injection of mesothelioma cells. This orthotopic model was compared with the most frequently used models where tumor cells were injected in the peritoneal cavity or subcutaneously. Tumor burden was quantified by in vivo imaging and histological confirmation, or tumor weight (intraperitoneal growth) or caliper (subcutaneous growth). Characterization of the leukocyte infiltrate (macrophages, lymphocytes) was performed by flow cytometry and immunohistochemistry.

As expected, each of the three tumor cell lines growing subcutaneously had a linear growth over time (with different kinetics), and tumors were easily measured by caliper at the end of the experiments; however, the subcutaneous growth does not represent an appropriate model for mesothelioma, since it is not an orthotopic model for MPM development. In the intraperitoneal model, we experienced some drawbacks; for instance, we noticed heterogeneous results of tumor growth among mice belonging to the same experiment, as quantified by luminescent signal in in vivo imaging and also observed at autopsy. Once injected in the peritoneum, mesothelioma cells formed several small tumors growing all over the surface of the abdominal organs. These masses were difficult to collect for a precise quantification of the tumor burden (as tumor weight). In some cases, tumor implantation caused an intestinal block and/or hemorrhagic ascites: these mice became suddenly sick and had to be sacrificed to avoid further suffering. We also noted that some mice died without having large tumor masses (as highlighted by autopsy analysis), but rather severe involvement of vital organs. Therefore, the parameter of mouse survival, which is usually employed in this i.p. model, can be affected, at least in some instances, independently of tumor progression. On one hand, the subcutaneous growth of MPM is not suitable to study MPM, since it is not an orthotopic model; on the other hand, in the intraperitoneal model, tumor cells invade not only the mesothelial lining but also the intraperitoneal organs, making this model unpredictable.

Therefore, we have set up an intrapleural orthotopic model with a minimally invasive procedure, injecting tumor cells in the intrathoracic space (between the third and fourth ribs). This model was fully characterized, verified for tumor growth by non-invasive optical in vivo imaging, and confirmed by histological examinations. Tumors developed along the internal surface of the pleura, further spreading and colonizing the most peripheral areas of the lungs, without forming any neoplastic mass outside the thoracic cavity. In vivo tumor growth was repeatedly monitored by acquiring a bioluminescent signal from luciferase-transduced cells over time. Our experiments revealed that tumor burden quantification by bioluminescence was totally trustable, and importantly, it was almost perfectly correlated with the quantification of tumor areas detected with conventional histology.

Mesothelioma mouse models in immunocompetent mice allow to study the involvement of immune cells and their relationship with tumor cells, which is of particular importance in the inflammation-driven carcinogenesis process of MPM. The characterization of the leukocyte infiltrate in the tumors was performed by flow citometry (for tumors grown s.c. or i.p.) and by immunohistochemistry on lung sections in the intrathoracic tumor model. The results were consistently homogeneous and revealed that murine mesothelioma are rich in infiltrating myeloid cells (mainly macrophages). This high content of tumor-associated macrophages well reflects the inflammatory micro-environment that characterizes also human MPM [16,17,18,19]. One tumor cell line (AB12) was also rich in T lymphocytes, which is an interesting finding in consideration of a potential response to checkpoint-based immunotherapy treatments, currently under clinical investigation in patients [7,20,21].

Few other intrathoracic models have been described in the literature, using either lung cancer cells or mesothelioma cells in rats or mice [13]. In some models, the surgical procedures performed were more invasive, employing a thoracotomy, which may be associated with appreciable mortality [22,23,24,25,26,27,28]. Instead, our model represents a minimally invasive procedure, consisting of a thin skin incision and a small needle puncture.

In some studies, human mesothelioma cells were injected in immunodeficient mice [13,29,30]. While the use of human cells is appreciable for a translation relevance, the tumor graft was below 70%, while in our experience the injection of murine tumor cells in syngeneic mice was 100% (in 50 mice). Furthermore, the use of immunodeficient mice, as mentioned above, cannot provide information on the role of the immune system in disease progression and in the anti-tumor response to conventional or immunotherapeutic treatments [16,19,31,32].

## 4. Materials and Methods

### 4.1. Cell Lines

AB1, AB12, and AB22 cells are deposited in the Australian cell bank and were kindly provided by Dr. M. Bianchi, San Raffaele Hospital, Milan, Italy [10,11]. AB1, AB12, and AB22 were cultured in RPMI (Roswell park memorial institute) 1640 (Lonza) supplemented with 10% FBS (fetal bovine serum) (Sigma), 2 mM L-glutamine, 100 U/mL penicillin and 100 μg/mL streptomycin (Life Technologies Inc.). All cell lines were cultured at 37 °C and 5% CO2 and routinely checked for mycoplasma contamination.

### 4.2. Mice

Mice were used in compliance with national (D.L. N. 26, G.U. 4 March 2014) and international law and policies (EEC Council Directive 2010/63/EU, OJ L 276/33, 22-09-2010; National Institutes of Health Guide for the Care and Use of Laboratory Animals, US National Research Council, 2011). BALB/c mice 8 weeks old were purchased from Charles River.

### 4.3. Injection of MPM Cells In Vivo in Mice: Set Up of an Intrathorax Model

BALB/c mice were anesthetized with ketamine/xylazine and shaved on the thoracic area with an electric shaver. Then, mice were placed in the left lateral decubitus position, and the thoracic wall was sterilized with 70% ethanol. An 8–10 mm skin incision were performed on the right thorax (close to the axillary cavity), and 5 × 10^4^ cells (resuspended in 50 uL of saline solution) were injected between the third and the fourth costal space, perpendicularly to the rib cage. A 29-gauge needle of a 500 ul syringe (U100, BD Becton, Dickinson) was used. To standardize the injection and avoid lung perforation, the needle was overmounted with a 200 ul tip, which was properly cut to expose only 3 mm of the needle. After cell injection, mice were then sutured, put back into a clean cage, and kept under a heating lamp (avoiding direct light) to recover from the anesthesia. Tumor growth quantification was performed by in vivo imaging over time.

In some experiments, tumor cells (1 × 10^5^ cells) were injected subcutaneously or intraperitoneally in BALB/c mice following standard procedures.

### 4.4. In Vivo Imaging

AB1, AB12, and AB22 murine mesothelioma cells have been previously infected with luciferase gene [11]. To evaluate the in vivo tumor growth over time, mice were i.p. injected with D-Luciferin (XenoLight D-Luciferin-K+ Salt, PerkinElmer; 150 mg Luciferin/kg body weight). Ten minutes after D-Luciferin injection, the bioluminescent signal was acquired using the IVIS Lumina III system (Perkin Elmer, Waltham, MA, USA). During the acquisition procedure, mice were anesthetized with Isoflurane (XGI-8 Gas anesthesia system, Perkin Elmer). Data were analyzed with Living image 4.3.1 by designing an ROI on the thoracic area of each mouse.

### 4.5. Flow Cytometry

The leukocyte infiltrate analysis of murine AB1, AB12, and AB22 mesothelioma was done with the following mAbs: CD45 PerCP (BD Biosciences, Eysins, Switzerland), CD11b Pacific Blue (Biolegend, San Diego, CA, USA), CD3 BV786 (BD Biosciences), CD4 FITC (eBioscience, San Diego, CA, USA), CD8 PE-Cy7 (BD Biosciences), F480 APC (AbD Serotec), Ly6G PE-Cy7 (BD Biosciences), and Ly6C FITC (BD Biosciences). Labeled cells were fixed in PBS (phosphate buffered saline) without calcium and magnesium 1× 1% formalin. Acquisition was performed at FACS Fortessa (BD Biosciences) and analyzed by FACS Diva and FlowJo software version 6.1.1 (BD Biosciences).

### 4.6. Immunohistochemistry

Paraffin-embedded murine tissues were cut at 3 um and placed on super-frost slides. After dewaxing and rehydration, antigen unmasking was performed with Decloaking Chamber in DIVA Buffer 1X (DV2005L2J Biocare Medical, Pacheco, CA, USA) (3 min at 125 °C, 5 min at 90 °C) (CD31, CD3, Ly6G); for IBA1 staining, antigen unmasking was not performed. Endogenous peroxidases were blocked with 2% H_2_O_2_ for 20 min and then rodent block M (for IBA1) or PBS/BSA (bovin serum albumin) 2% (for CD31, CD3, and Ly6G staining) were used to block unspecific binding sites. Sections were incubated with the following antibodies: rabbit anti-mouse IBA-1 (1:250, Wako), goat anti-mouse CD31 (1:1000, R&D), rat anti-mouse CD3 (1:1000, Serotec), and rat anti-mouse Ly6G (1:200, BD Biosciences). All the primary antibodies were incubated for 1 h in a humid chamber at room temperature. As secondary antibody, we used a Rat on Mouse HRP polymer kit (Biocare Medical) (CD3, Ly6G), Goat on Rodent (Biocare Medical) (CD31), and Mach1 (Biocare Medical) (IBA1). Reactions were developed with 3,3′-diaminobenzidine, DAB (Biocare Medical) and then counterstained with hematoxylin and mounted with Eukitt.

## 5. Conclusions

We have set up and characterized an orthotopic model of pleural mesothelioma in mice. Advantages of our model include the following: the procedure to inject mesothelioma tumor cells in the thorax is minimally invasive; mesothelioma cells grow along the external surface of the lungs in 100% of mice; in vivo quantification of tumor growth by bioluminescence is linear along time and reliably matches the histological examinations. This model has been successful with three different murine cell lines implanted in immunocompetent mice, with no complication of tumor rejection due to luciferase-expressing cells. Overall, our intrathoracic model of murine mesothelioma is simple and reproducible, and it faithfully recapitulates the growth pattern of human MPM.

## Supplementary Materials

The following are available online at https://www.mdpi.com/2072-6694/12/8/2136/s1, Figure S1: Gating strategy of flow cytometry. Physical parameters were used to sequentially exclude debris and doublets from the analysis. On singlets, live cells were defined as BV515 negative events. Leukocyte infiltrate was defined on the basis of CD45 expression. CD11b was used to identify lymphoid (CD11b^−^) and myeloid (CD11b^+^) cells. In the lymphoid compartment we identified CD4^+^ lymphocytes (CD45^+^/CD11b^−^/CD3^+^/CD4^+^ cells) and CD8^+^ lymphocytes (CD45^+^/CD11b^−^/CD3^+^/CD8^+^ cells). In the myeloid compartment Macrophages were defined as CD45^+^/CD11b^+^/Ly6C^low^/F4/80^+^ cells, monocytes as CD45^+^/CD11b^+^/Ly6C^high^/LY6G^low^/F4-80^low^, neutrophils were defined as CD45^+^/ CD11b^+^/LY6C^low^/LY6G^+^/F4-80^−^ cells, Figure S2: Average radiance of AB1, AB12 and AB22 injected mice (Intra-thoracically). Raw data about the experiment shown in Figure 5. Average radiance is measured in p/s/cm^2^/sr.
