# Supplementary material for: Optimization of a Luciferase-Expressing Non-Invasive Intrapleural Model of Malignant Mesothelioma in Immunocompetent Mice
Source: Cancers (Basel). 2020 Aug 1;12(8):2136. doi: 10.3390/cancers12082136 (PMC7465989; doi:10.3390/cancers12082136)
Supplement: Supplementary file 1 [file cancers-12-02136-s001.pdf]

Supplementary Materials: Optimization of a luciferase-expressing non-invasive intra-pleural model of malignant mesothelioma in immunocompetent mice

Elisabeth Digifico, Marco Erreni, Federico Simone Colombo, Camilla Recordati, Roberta Migliore, Roberta Frapolli, Maurizio D'Incalci, Cristina Belgiovine and Paola Allavena

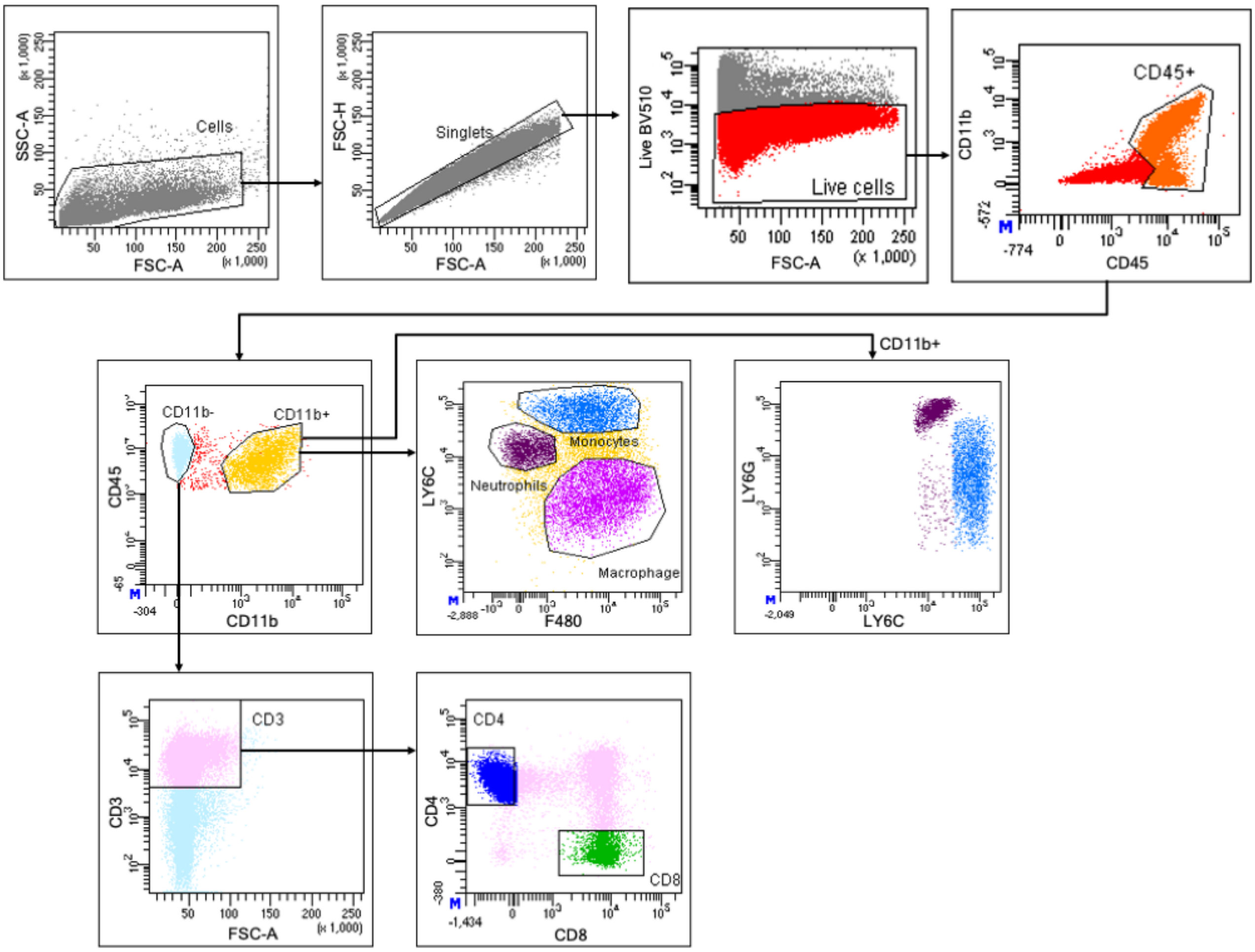

Figure S1. Gating strategy of flow cytometry. Physical parameters were used to sequentially exclude debris and doublets from the analysis. On singlets, live cells were defined as BV515 negative events. Leukocyte infiltrate was defined on the basis of CD45 expression. CD11b was used to identify lymphoid (CD11b^{-}) and myeloid (CD11b^{+}) cells. In the lymphoid compartment we identified CD4^{+} lymphocytes (CD45^{+}/CD11b^{-}/CD3^{+}/CD4^{+} cells) and CD8^{+} lymphocytes (CD45^{+}/CD11b^{-}/CD3^{+}/CD8^{+} cells). In the myeloid compartment Macrophages were defined as CD45^{+}/CD11b^{+}/Ly6C^{low}/F4/80^{+} cells, monocytes as CD45^{+}/CD11b^{+}/Ly6C^{high}/LY6G^{low}/F4-80^{low}, neutrophils were defined as CD45^{+}/ CD11b^{+}/LY6C^{low}/LY6G^{+}/F4-80^{-} cells.

	AB1 (Average Radiance)				
Days	Mouse 1	Mouse 2	Mouse 3	Mouse 4	Mouse 5
4	33600	17200	1100	28500	1160
6	86000	89700	5320	44100	3700
8	103000	161000	4780	54700	9860
11	47800	112000	1500	18700	62900
**14**	**151000**	**220000**	**3140**	**44500**	**108000**

	AB12 (Average Radiance)				
Days	Mouse 1	Mouse 2	Mouse 3	Mouse 4	Mouse 5
7	29200	38900	10400	1060	37000
12	493000	629000	13400	183000	498000
**16**	**1130000**	**1240000**	**783000**	**357000**	**973000**

	AB22 (Average Radiance)				
Days	Mouse 1	Mouse 2	Mouse 3	Mouse 4	Mouse 5
3	4560	1750	5180	7070	10500
9	3380	5920	37300	69200	1730
**16**	**52800**	**169000**	**382000**	**1140000**	**685000**

Figure S2. Average radiance of AB1, AB12 and AB22 injected mice (Intra-thoracically). Raw data about the experiment shown in Figure 5. Average radiance is measured in p/sec/cm^2/sr.

© 2020 by the authors. Licensee MDPI, Basel, Switzerland. This article is an open access article distributed under the terms and conditions of the Creative Commons Attribution (CC BY) license (http://creativecommons.org/licenses/by/4.0/).
